# Coumarin *N*-Acylhydrazone Derivatives: Green Synthesis and Antioxidant Potential—Experimental and Theoretical Study

**DOI:** 10.3390/antiox12101858

**Published:** 2023-10-13

**Authors:** Dušica M. Simijonović, Dejan A. Milenković, Edina H. Avdović, Žiko B. Milanović, Marko R. Antonijević, Ana D. Amić, Zana Dolićanin, Zoran S. Marković

**Affiliations:** 1 Department of Science, Institute for Information Technologies, University of Kragujevac, Jovana Cvijića bb, 34000 Kragujevac, Serbia; dusicachem@kg.ac.rs (D.M.S.); edina.avdovic@pmf.kg.ac.rs (E.H.A.); ziko.milanovic@uni.kg.ac.rs (Ž.B.M.); mantonijevic@uni.kg.ac.rs (M.R.A.); 2 Department of Chemistry, Josip Juraj Strossmayer University of Osijek, Ulica Cara Hadrijana 8A, 31000 Osijek, Croatia; aamic@kemija.unios.hr; 3 Department of Biomedical Sciences, State University of Novi Pazar, Vuka Karadžića bb, 36300 Novi Pazar, Serbia; zdolicanin@np.ac.rs; 4 Department of Natural Science and Mathematics, State University of Novi Pazar, Vuka Karadžića bb, 36300 Novi Pazar, Serbia

**Keywords:** coumarin *N*-acylhydrazone derivatives, green synthesis, antioxidant activity, DFT

## Abstract

Coumarin *N*-acylhydrazone derivatives were synthesized in the reaction of 3-acetylcoumarin and different benzohydrazides in the presence of molecular iodine as catalyst and at room temperature. All reactions were rapidly completed, and products were obtained in good to excellent yields. It is important to emphasize that four products were reported for the first time in this study. The obtained compounds were subjected to evaluation of their in vitro antioxidative activity using DPPH, ABTS, and FRAP methods. It was shown that products with a catechol moiety in their structure are the most potent antioxidant agents. The thermodynamic parameters and Gibbs free energies of reactions were used to determine the most probable mechanism of action. The results of in silico examination emphasize the need to take solvent polarity and free radical species into account when examining antiradical action. It was discovered by using computational approaches that HAT and SPLET are competitive molecular pathways for the radical scavenging activity of all compounds in polar mediums, while the HAT is the dominant mechanism in non-polar environments.

## 1. Introduction

**Figure 1:**
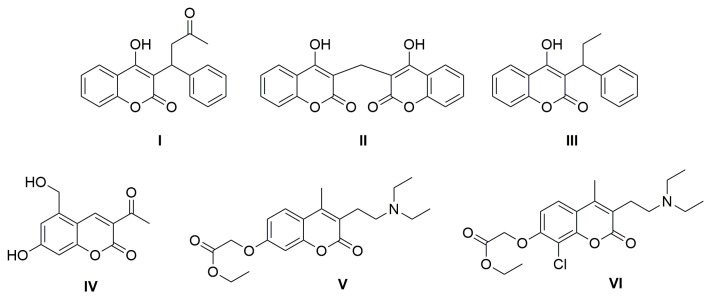
Structures of some coumarin derivatives with clinical application.

Coumarins constitute a large group of natural compounds that are extensively prevalent in nature [1,2]. In addition to fruits, they are most commonly found in the roots, stems, and leaves of higher plants. Some essential oils, such as lavender oil, cinnamon bark oil, and cassia leaf oil, contain large amounts of different coumarin derivatives. Certain varieties of cinnamon are the most common source of coumarins for humans [3]. Coumarins are relatively simple compounds that basically consist of fused α-pyrone and a benzene ring. Coumarins that occur naturally exhibit a wide range of pharmacological activities and are frequently employed by researchers in the design and development of novel synthetic coumarins [4]. The significance of coumarin hybrids in the fields of medicine and pharmacy is noteworthy due to their relatively low level of adverse effects on living things, as reported in the literature [5]. Several pharmacological activities, such as antioxidant [6,7,8], anticancer [9,10], antitubercular [11], antibacterial [12,13,14], antifungal [15], antiviral [16], and anticoagulant [17,18,19,20,21], have been exhibited by many of the obtained hybrid compounds. Chemically modified coumarins have great potential as drugs for the treatment of various diseases. Several coumarin derivatives, including warfarin (I), dicoumarol (II), phenprocoumon (III), armilarizine A (IV), carbochromen (V) and chlorochromen (VI), have found significant clinical application, Figure 1.

Hydrazone compounds are a well-known group of compounds with specific structural units (R_2_C=N–NH_2_). These compounds are of great significance in organic synthesis and in the creation of compounds that exhibit a wide range of biological activities. The literature describes the coumarin compounds with various hydrazide–hydrazone pharmacophores attached to position three in the chromen ring [22,23,24,25,26]. The synthesis of coumarin hydrazone derivatives involves the condensation of diverse 3-formylcoumarin with different hydrazides in the presence of acid as a catalyst [22,23]. According to the literature, a method is known for the synthesis of a series of 3-acylhydrazono-4-hydroxycoumarins using the reaction of 3-acetyl-4-hydroxycoumarin with the corresponding hydrazides [24,25,26]. However, it is worth pointing out that the reactions between 3-acetylcoumarin and various benzoyl hydrazides have been described in only one article [27]. Pangal et al. conducted a study wherein they successfully synthesized three derivatives of coumarin *N*-acylhydrazone through the reaction of 3-acetylcoumarin with appropriate hydrazides under reflux in methanol.

In this paper, we report a green methodology for the synthesis of coumarin *N*-acylhydrazone derivatives in ethanol and in the presence of molecular iodine as an efficient, relatively nontoxic, and inexpensive catalyst. Additionally, this study involves the assessment of the antioxidative potential of the obtained products by using both experimental and theoretical approaches.

## 2. Materials and Methods

All chemicals used for the synthesis were purchased from Sigma Aldrich, Darmstadt, Germany with purities above 98%. The IR spectra were obtained on a PerkinElmer Spectrum One FT-IR spectrometer using the KBr disc. The NMR spectra were recorded on a Varian Gemini spectrometer (200 MHz for ^1^H and 50 MHz for ^13^C) by using DMSO-*d*_6_ as solvent. All UV-Vis determinations were carried out using a PerkinElmer, Lambda 365 UV/Vis Spectrophotometer. Elemental microanalysis for carbon, hydrogen, and nitrogen was performed at the Institute for Information Technologies, University of Kragujevac. Elemental (C, H, N) analysis of the samples was carried out on an Elemental analysis system NC Technologies, ECS 8020 CHNOS, model-dual furnace-NC.

### 2.1. Synthesis of Coumarin N-Acylhydrazone Derivatives

Coumarin *N*-acylhydrazone derivatives **3** were obtained in the reaction of 3-acetylcoumarin (0.001 mol) and corresponding benzohydrazide **2** (0.001 mol) in presence of molecular iodine as catalyst (10 mol%) in absolute ethanol at room temperature. The progress of the reaction was monitored using thin-layer chromatography (TLC). Upon completion of the reaction, the purified product was isolated by filtration. All coumarin *N*-acylhydrazone derivatives (**3a**–**f**) were characterized with ^1^H NMR, ^13^C NMR, IR, and UV-Vis spectra. In addition, elemental analysis confirmed the purity of the newly synthesized compounds **3b**–**f**.

(*E*)-4-hydroxy-*N′*-(1-(2-oxo-2*H*-chromen-3-yl)ethylidene)benzohydrazide (**3a**): ^1^H NMR (200 MHz, DMSO-*d*_6_) δ 10.57 (s, 1H), 10.11 (s, 1H), 8.22 (s, 1H), 7.88 (dd, *J* = 7.8, 1.5 Hz, 1H), 7.83–7.74 (m, 2H), 7.66 (ddd, *J* = 8.7, 7.2, 1.7 Hz, 1H), 7.50–7.34 (m, 2H), 6.91–6.80 (m, 2H), 2.32 (s, 3H); ^13^C NMR (50 MHz, DMSO-*d*_6_) δ 160.7, 159.3, 153.5, 141.5, 132.4, 130.3, 129.2, 127.0, 124.8, 124.7, 118.9, 116.1, 114.9, 16.7; IR (KBr): 3282 (OH), 3205 (NH), 3040 (ArC-H), 3018 (CH), 1707, 1659 (C=O), 1276 (C–N), 1222, 1163 (C–O) cm^−1^; UV (λ_max_): 269, 327 nm.

(*E*)-2-hydroxy-*N′*-(1-(2-oxo-2*H*-chromen-3-yl)ethylidene)benzohydrazide (**3b**): ^1^H NMR (200 MHz, DMSO-*d*_6_) δ 11.79 (s, 1H), 11.41 (s, 1H), 8.28 (s, 1H), 7.94 (dd, *J* = 20.2, 7.8 Hz, 2H), 7.67 (m, *J* = 8.7, 7.3, 1.6 Hz, 1H), 7.46 (d, *J* = 8.4 Hz, 2H), 7.38 (d, *J* = 7.7 Hz, 1H), 7.07–6.85 (m, 2H), 2.30 (s, 3H); ^13^C NMR (50 MHz, DMSO-*d*_6_) δ 159.3, 156.5, 153.5, 141.7, 133.5, 132.5, 130.7, 129.3, 124.9, 119.7, 118.8, 116.9, 116.1, 15.8; IR (KBr): 3435 (OH), 3262 (NH), 3051 (ArC-H), 2928 (CH), 1725, 1691 (C=O), 1299 (C–N), 1161 (C–O) cm^−1^; UV (λ_max_): 267, 330 nm.

(*E*)-3,4-dihydroxy-*N′*-(1-(2-oxo-2*H*-chromen-3-yl)ethylidene)benzohydrazide (**3c**): ^1^H NMR (200 MHz, DMSO-*d*_6_) δ 10.53 (s, 1H), 9.61 (s, 1H), 9.29 (s, 1H), 8.22 (s, 1H), 7.95–7.55 (m, 2H), 7.52–7.12 (m, 4H), 6.82 (d, *J* = 8.1 Hz, 1H), 2.31 (s, 3H); ^13^C NMR (50 MHz, DMSO-*d*_6_) δ 159.3, 153.5, 149.1, 145.0, 141.5, 132.4, 129.2, 124.8, 124.7, 120.3, 118.9, 116.1, 115.0, 16.2; IR (KBr): 3429 (OH, NH), 3077 (ArC-H), 2929 (CH), 1712, 1698 (C=O), 1309 (C–N), 1220 (C–O) cm^−1^; UV (λ_max_): 271, 328 nm; C_18_H_14_N_2_O_5_ (FW = 338.32): C, 63.90; N, 8.28; H, 4.17%; found: C, 64.27; N, 8.15; H, 4.52%.

(*E*)-2,3-dihydroxy-*N′*-(1-(2-oxo-2*H*-chromen-3-yl)ethylidene)benzohydrazide (**3d**): ^1^H NMR (200 MHz, DMSO-*d*_6_) δ 11.46 (s, 1H), 10.88 (s, 1H), 9.86 (s, 1H), 8.27 (s, 1H), 7.98–7.58 (m, 2H), 7.52–7.15 (m, 3H), 7.08–6.59 (m, 2H), 2.28 (s, 3H); ^13^C NMR (50 MHz, DMSO-*d*_6_) δ 159.3, 153.5, 146.1, 141.7, 132.6, 129.3, 124.9, 120.2, 119.3, 118.9, 118.8, 116.1, 15.9; IR (KBr): 3502, 3430 (OH), 3250 (NH), 3064 (ArC-H), 2873 (CH), 1690, 1649 (C=O), 1283 (C–N), 1140 (C–O) cm^−1^; UV (λ_max_): 266, 333 nm; C_18_H_14_N_2_O_5_ (FW = 338.32): C, 63.90; N, 8.28; H, 4.17%; found: C, 63.67; N, 8.35; H, 4.56%.

(*E*)-3,4,5-trimethoxy-*N′*-(1-(2-oxo-2*H*-chromen-3-yl)ethylidene)benzohydrazide (**3e**): ^1^H NMR (200 MHz, DMSO-*d*_6_) δ 10.75 (s, 1H), 8.24 (s, 1H), 7.88 (d, *J* = 7.3 Hz, 1H), 7.73–7.59 (m, 1H), 7.41 (dd, *J* = 15.3, 7.9 Hz, 2H), 7.20 (s, 2H), 3.86 (s, 6H), 3.73 (s, 3H), 2.34 (s, 3H); ^13^C NMR (50 MHz, DMSO-*d*_6_) δ 159.2, 153.5, 152.6, 132.5, 129.3, 129.0, 126.9, 124.8, 118.82, 116.1, 105.9, 60.2, 16.5; IR (KBr): 3428 (OH), 3277 (NH), 3048 (ArC-H), 2910 (CH), 1719, 1665 (C=O), 1230 (C–N), 1127 (C–O) cm^−1^; UV (λ_max_): 276, 329 nm; C_21_H_20_N_2_O_6_ (FW = 396.40): C, 63.63; N, 7.07; H, 5.09%; found: C, 63.87; N, 6.85; H, 4.72%.

(*E*)-4-hydroxy-3-methoxy-*N′*-(1-(2-oxo-2*H*-chromen-3-yl)ethylidene)benzohydrazide (**3f**): ^1^H NMR (200 MHz, DMSO-*d*_6_) δ 10.59 (s, 1H), 9.73 (s, 1H), 8.22 (s, 1H), 7.87 (dd, *J* = 7.7, 1.4 Hz, 1H), 7.66 (ddd, *J* = 8.8, 7.3, 1.6 Hz, 1H), 7.51–7.33 (m, 4H), 6.87 (d, *J* = 8.3 Hz, 1H), 3.85 (s, 3H), 2.33 (s, 3H); ^13^C NMR (50 MHz, DMSO-*d*_6_) δ 159.3, 153.5, 150.2, 141.5, 132.4, 129.2, 127.0, 124.8, 124.5, 122.1, 118.9, 116.1, 114.9, 55.9, 16.4; IR (KBr): 3428 (OH), 3337 (NH), 3078 (ArC-H), 2941 (CH), 1712, 1649 (C=O), 1280 (C–N), 1121 (C–O) cm^−1^; UV (λ_max_): 269, 324 nm; C_19_H_16_N_2_O_5_ (FW = 352.35): C, 64.77; N, 7.95; H, 4.58%; found: C, 64.97; N, 7.79; H, 4.53%. 

### 2.2. DPPH Radical Scavenging Assay

The free radical scavenging activities of referent compounds and synthesized products were determined using a 1,1-diphenyl-2-picryl-hydrazyl (DPPH) assay [28]. The tested samples (20 µL of different concentrations dissolved in DMSO and 980 µL of methanol) were mixed with the same volume of the solution of DPPH in methanol (0.05 mM). The prepared samples were shaken well and left at room temperature in the dark for 20 min and 60 min. After the incubation period, absorbance was determined at 517 nm by using the methanol as a blank control. All tests were run in triplicate and averaged. The results are presented as mean value ± standard deviation. Nordihydroguaiaretic acid (NDGA) and quercetin were used as positive controls. For products that exert good activity, IC_50_ values were determined, and the corresponding concentrations used for this determination are given in Table S1. The stoichiometric factor (*SF*) was calculated using next equation:

$$SF=\frac{{\left[DPPH\right]}_{0}}{\left(2\times IC_{50}\right)}.$$

### 2.3. ABTS Radical Cation Scavenging Assay

The antioxidant capacity was measured using the 2,2′-azino-bis(3-ethylbenzothiazoline-6-sulfonic acid) diammonium salt) (ABTS) assay according to the modified procedure of Pontiki et al. [29]. In this assay, stock solutions of ABTS (7 mM) and potassium persulfate (2.45 mM) were first prepared. The working solution was then prepared by mixing the two stock solutions in equal quantities and allowing them to react for 12–16 h at room temperature in the dark. The ABTS^+•^ radical cation is diluted with methanol to obtain the solution with an absorbance of 0.70 units at 734 nm. Different concentrations of samples were prepared in DMSO. Next, 20 μL of sample and 980 μL of methanol were mixed, then an equal amount of ABTS was added and the absorbance was measured at 734 nm. IC_50_ values were determined for products with noticeable activity and the concentrations used for this determination are given in Table S2.

### 2.4. Ferric Ion Reducing Capacity Assay

The ferric ion reducing capacity (FRAP) assay is a direct method for measuring the combined antioxidant activity of reductive antioxidants in a substance under investigation. In this study, the FRAP assay was conducted with some modifications to the method described by Pownall et al. [30]. The stock solutions of tested compounds were prepared in DMSO, and then sample dilutions were prepared in phosphate buffer, pH 7.4. After that, 500 µL of diluted samples were mixed with 250 µL of 1% potassium ferricyanide solution followed by incubation for 20 min at 50 °C. Following the incubation period, a mixture was prepared by combining 500 µL of the sample with 500 µL of 10% trichloroacetic acid, 100 µL of 0.1% ferric chloride, and 500 µL of deionized water. The mixture was subjected to an incubation period of 10 min at room temperature, after which, the absorbance was measured at the wavelength of 700 nm. Concentrations of investigated compounds and referent standard ascorbic acid in the sample were 10 µM. The results of reducing the power of compounds were expressed as % Activity of Ascorbic acid (% *AAa*) [31].

$$\%\;AAa=\frac{A_{sample}}{A_{reference}}\times 100$$

### 2.5. Computational Methodology

The Gaussian09 software package was used to carry out all calculations [32]. Quantum chemical calculations based on density functional theory, specifically M06-2X functional in conjunction with 6-311++G(d,p) basis set (with polarization and diffuse functions included), were used to optimize the structures of parent molecules as well as of the corresponding radicals, anions, and radical cations [33,34]. Previous research has demonstrated that the M062-X method is suitable for thermodynamic and kinetic investigation of reaction mechanisms of examined compounds with free radicals and that it describes short- and medium-range interatomic interactions well (500 pm) [35]. The SMD continuum solvation model [36] was used to simulate the structures in benzene (ε = 2.27) and methanol (ε = 32.61) with no geometrical restrictions. These solvents were chosen to replicate both polar and nonpolar settings as well as the environment in which experimental measurements were conducted.

For the assessment of the antioxidant activity, three main radical scavenging mechanisms—Hydrogen Atom Transfer (HAT), Single-Electron Transfer Followed by Proton Transfer (SET-PT), and Sequential Proton Loss-Electron Transfer (SPLET) were used [37,38,39,40]. In the first reaction mechanism, the O–H bond is homolytic cleavage, resulting in the separation of a hydrogen atom and a radical species (A–O^•^) (Equation (1)). In the first step of the SET-PT mechanism, a molecule loses an electron, which leads to the formation of the radical cation (A–OH^+•^), which loses a proton in the second step (Equations (2) and (3)). Another two-step mechanism is the SPLET mechanism. The antioxidant molecule is used to create an anion (A–O^−^) in the first stage, and the matching radical is created in the second step following an electron transfer (Equations (4) and (5)).

A–OH → A–O^•^ + H^•^ (1)

A–OH → A–OH^+•^ + e^−^ (2)

A–OH^+•^ → A–O^•^ + H^+^ (3)

A–OH → A–O^−^ + H^+^ (4)

A–O^−^ → A–O^•^ + e^−^ (5)

BDE (Bond Dissociation Enthalpy, Equation (6)), IP (Ionization Potential, Equation (7)), and PDE (Proton Dissociation Enthalpy, Equation (8)), as well as PA (Proton Affinity, Equation (9)) and ETE (Electron Transfer Enthalpy, Equation (10)), are the thermodynamic parameters driving the antioxidative mechanisms. The following equations were used to calculate the parameters, at 298.15 K, from the enthalpies of the optimized species [41,42].

BDE = *H*(A–O^•^) + *H*(H^•^) − *H*(A–OH) (6)

IP = *H*(A–OH^•+^) + *H*(e^−^) − *H*(A–OH) (7)

PDE = *H*(A–O^•^) + *H*(H^+^) − *H*(A–OH^•+^) (8)

PA = *H*(A–O^−^) + *H*(H^+^) − *H*(A–OH) (9)

ETE = *H*(A–O^•^) + *H*(e^−^) − *H*(A–O^−^) (10)

The solvated electron and proton enthalpies for the M062-X technique in benzene (−877.4 and −10.5 kJ mol^−1^) and methanol (−1065.4 and −61.4 kJ mol^−1^) were obtained from the available data in the literature [43]. The Natural Bond Orbital (NBO) method and NBO 6.0 package were used to conduct the population study [44]. The intermolecular orbitals’ role in the examined compounds’ anions and radicals is highlighted using NBO analysis.

## 3. Results and Discussion

**Scheme 1:**
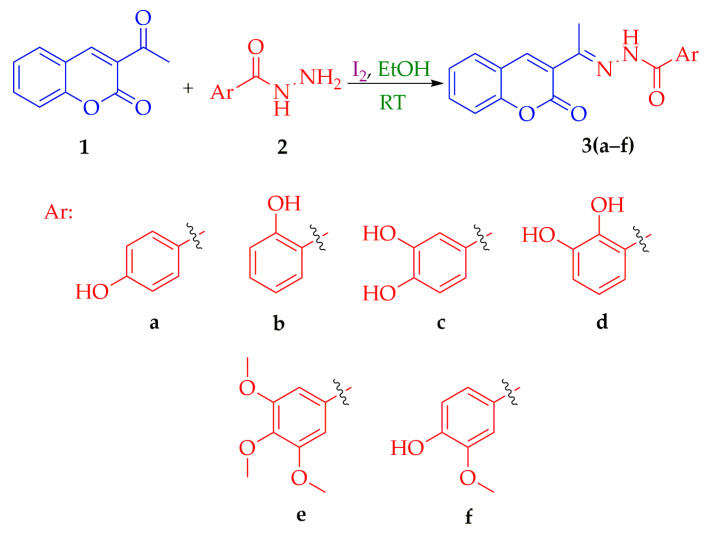
Synthesis of coumarin *N*-acylhydrazone derivatives: 3-acetylcoumarin (**1**), benzoyl hydrazide (**2**), iodine as catalyst (10 mol%), absolute ethanol, and room temperature (RT).

Our recent results related to the preparation of coumarin-hydroxybenzohydrazide derivatives prompted us to carry out the synthesis of structurally similar compounds starting from 3-acetylcoumarin [26]. The synthesis of various coumarin hydrazones (**3**) from 3-acetylcoumarin (**1**) and the corresponding benzoyl hydrazide (**2**) was presented in Scheme 1. The reaction between **1** and 4-hydroxybenzohydrazide (**2a**) in ethanol was used as a model for optimizing reaction conditions (Table 1). Initially, the reaction was conducted at room temperature and without a catalyst, but only trace amounts of the reaction product were obtained. The moderate yield of the product was observed after 10 h of heating the reaction mixture under reflux. In order to attain a higher product yield, acetic acid was used as a catalyst. In this instance, an adequate yield of **3a** was obtained by increasing the catalyst concentration to 20 mol% and heating the mixture for 10 h. However, the catalyst had a substantial effect on both the reaction rate and yield. Specifically, the use of molecular iodine as a catalyst allowed the desired product **3a** to be obtained in only 20 min and without heating (Table 1). The optimal conditions for carrying out these reactions were 10 mol% I_2_ as a catalyst, room temperature, and 20 min.

The results indicate that the applied reaction conditions result in the outstanding dissolution of the reactants, rapid precipitation of the product, and simple isolation of the reaction products. Table 2 shows that all products were obtained with yields ranging from outstanding to very good. All reactions were completed very rapidly, except for compounds **3d** and **3e**, for which a slight extension of the reaction time was required. It is essential to note that four obtained products have been reported for the first time in this study (**3c**–**f**). The isolated products’ structures were characterized using NMR, IR, and UV-Vis spectroscopies. Additionally, elemental analysis was performed on newly synthesized compounds.

### 3.1. Structural Characterization of Coumarin N-Acylhydrazone Derivatives

The signals in the ^1^H NMR spectra of derivatives **3** were assigned to three groups of protons. Singlets originating from the protons of the methyl group were observed at approximately 2.30 ppm in the ^1^H NMR spectra of all the compounds. The aromatic protons of coumarin and hydrazide moiety were in a region of 6.64–8.01 ppm. The hydrogen atoms of NH and phenolic OH groups were observed in the regions of 10.53–11.79 ppm and 9.29–11.41 ppm as broad singlets. In the spectra of compounds **3e** and **3f**, singlets originating from protons of methoxy groups attached to the aromatic ring at about 3.85 ppm were observed. In the ^13^C NMR spectra of all the products, the carbon atom of the methyl group appears around 17 ppm. Signals in the range of 105–145 ppm were attributed to aromatic carbon atoms. Peaks detected at around 150, 153, and 160 ppm originate from C–O, C–N, and C=O carbon atoms. The additional peaks at about 60 ppm were observed in the spectra of compounds **3e** and **3f** because of the presence of methoxy groups.

The vibration of OH and NH groups corresponds to regions at approximately 3400 and 3250 cm^−1^ in the IR spectra of investigated compounds. The peaks in region 1650–1725 cm^−1^ are assigned to the C=O of hydrazone group and lactone ring, while bands present at lower values are attributed to the stretching vibration of C–N, C–O, and aromatic rings. In the UV-Vis spectra of all the compounds, two major absorption bands appear around 270 and 330 nm.

### 3.2. The Proposed Mechanism for the Synthesis of Coumarin N-Acylhydrazone Derivatives ***3***

**Scheme 2:**
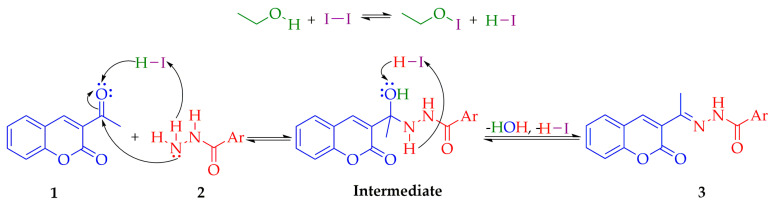
The suggested mechanism for formation of coumarin *N*-acylhydrazone derivatives.

The suggested mechanism for the synthesis of coumarin *N*-acylhydrazone derivatives **3** was presented in Scheme 2. In this reaction, molecular iodine most likely acts only as a precatalytic. In the first step, as a solvent, ethanol reacts with molecular iodine to give the Brønsted acid HI, which further acts as a catalyst, as shown in Scheme 2. In the subsequent step, the carbonyl group of 3-acetylcoumarin is protonated with Brønsted acid HI, followed by the nucleophilic attack of hydrazide’s nitrogen atom to the carbonyl group, and the deprotonation of the same nitrogen atom. In this manner, an appropriate hemiaminal is formed as an intermediate. The resulting intermediate undergoes dehydration in a similar way. During this stage, the HI species facilitates the protonation of the hydroxyl group, leading to the formation of a water molecule, with simultaneous deprotonation of the nitrogen atom. In this way, corresponding coumarin *N*-acylhydrazone derivative **3** is formed.

### 3.3. Antioxidant Activity of Coumarin N-Acylhydrazone Derivatives

The DPPH and ABTS scavenging ability, as well as the ferric ion reducing capacity, of the synthesized coumarin *N*-acylhydrazone derivatives (**3a**–**f**) were evaluated to determine their in vitro antioxidant properties. The results are presented in Table 3 and Table 4. In this study, NDGA, quercetin, (*S*)-6-methoxy-2,5,7,8-tetramethylchromane-2-carboxylic acid (Trolox), and ascorbic acid were used as referent compounds.

The results obtained from the DPPH assay revealed that compounds **3c** and **3d** expressed the best antioxidant activity with IC_50_ values of 2.1 µM and 3.2 µM. The activity of these compounds is slightly lower than the activity of the positive controls, quercetin and NDGA. In order to determine whether the most active compounds belong to the group of good antioxidants, the SF was determined [26,45]. It is known that compounds with an SF greater than 2 are good antioxidants. Namely, the tested compounds **3c** and **3d**, with SF values 6 and 3.9, belong to the group of excellent antioxidants. Other tested compounds showed extremely low activity toward the DPPH radical even at significantly higher concentrations, Table 3.

The ABTS assay, which is a commonly employed technique for evaluating in vitro antioxidant capacity, was utilized to examine the radical scavenging potential of the investigated compounds, as presented in Table 4. The results obtained from this study indicate that compounds **3c** and **3d** exhibit significant scavenging activity against ABTS radical cation, with IC_50_ values of 2.2 µM and 3.0 µM, respectively. This finding is consistent with previous DPPH assay results. Compound **3f** exhibited better activity relative to the DPPH results, exhibiting an efficacy of roughly 50% at a concentration of 100 µM.

The results of the FRAP assay revealed that all the examined compounds exhibited higher capacities for Fe^3+^ reduction than ascorbic acid, which was used as the standard. It should be emphasized that the obtained results are in good agreement with the activities of these compounds against DPPH and ABTS radicals. Namely, compound **3c** showed the greatest reducing ability, followed by compounds **3d** and **3f**. All the obtained results point out the compounds **3c** and **3d**, with two neighboring hydroxyl groups, i.e., compounds with a catechol fragment, as the best antioxidants. In addition, moderate activity against the ABTS radical cation is observed for compound **3f** with vanillic fragment. The reason for this is the resonance and electron donating effects of these groups, as well as the very effective stabilization of the formed phenoxy radical by an intramolecular hydrogen bond.

### 3.4. Determination of the Plausible Mechanisms

**Figure 2:**
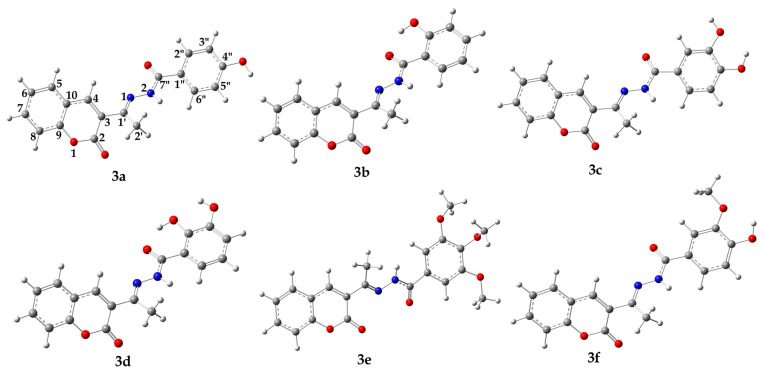
Optimized geometries of investigated compounds, **3a**–**3f** at M06-2X/6-311++G(d,p) level of theory in methanol (SMD solvation model). Legend: gray—carbon atom, white—hydrogen atom, red—oxygen atom, blue—nitrogen atom.

The optimized geometries of the newly synthesized compounds, as shown in Figure 2, were obtained using the M06-2X/6-311++G(d,p) theoretical model. The geometries of the compounds under investigation exhibited a correlation with the geometries of structurally analogous compounds obtained in previous research [26,46,47]. An examination of the optimal molecular structure of the six coumarin derivatives revealed the existence of a partly double N–N bond, which facilitated the delocalization of π-electrons between the coumarin base and the fused aromatic ring. Based on the observed value of the dihedral angle C2–C3–C1′–N1, which is around ~130°, it is apparent that all the structures display deviations from planarity. Moreover, the determined N2–C7″–C1″–C6″ dihedral angles within the range of 152–179° for all the examined compounds suggest a deviation from planarity and a reduction in the electronic distribution within the compounds, which significantly affects their antioxidant activity. The obtained geometries were used for a deeper study of the mechanisms of the antioxidant action.

#### 3.4.1. HAT Mechanism

The analysis of the BDE values for homolytic O–H cleavage showed that the reactivity decreased in the following order: **3d** > **3c** > **3f** > **3b** > **3a** > **3e** (Table 5). The reactivity of a compound is correlated with the stability of the newly formed radical species. Therefore, the NBO spin density of the studied radical species was analyzed. Figure S1 shows that the unpaired electron was mainly delocalized over the oxygen undergoing hydrogen abstraction and the ortho and para carbon atoms. Obviously, the better the delocalization of the unpaired electron, the more stable the obtained radical. The radicals formed from the *o*- and *p*-hydroxyl groups are the most stable because their unpaired electrons are delocalized over the benzene ring. The spin density values of the newly formed radicals increased in the following order: **3d-2O^•^** (0.284e) > **3f** (0.306e) > **3c-4O^•^** (0.312e) > **3b** (0.327e) > **3d-3O^•^** (0.330e) > **3c-3O^•^** (0.341e) > **3a** (0.358e). The oxygen atom of the strongest 3d radical scavenger has the lowest spin density. The lower BDE value for **3d** can be attributed to the stability of the radical species due to the free rotation of the C7″–C1″ bond, as well as the formation of an intramolecular hydrogen bond. Since the BDE values in benzene are comparatively lower than the PA and ETE values, it can be concluded that HAT is likely to be the dominant mechanism in nonpolar solvents. 

On the other hand, the calculated BDE values for the homolytic cleavage of N–H bonds exhibited an increasing trend in the following order: **3e** > **3b** > **3d**> **3f** > **3c** > **3a**. The limited delocalization of the electron and the reduced stability of radicals arising from the homolytic cleavage of the N2–H bond can be attributed to the higher spin density values observed for N-centered radicals compared with O-centered radicals (Figure S2). This discrepancy is exemplified by the higher BDE values associated with N-centered radicals. 

Generally, the BDE values obtained for compounds **3a**–**f** exhibit similarities to the BDE values reported for the conventional antioxidants apigenin (372 and 368 kJ mol^−1^) and naringenin (368 and 362 kJ mol^−1^) [48]. The BDE values show a noticeably lower value in the non-polar solvent relative to the values in methanol, as shown in Table 5. These results are expected and in good agreement with the results of earlier research [26,36,37,38,39,49,50,51,52,53].

#### 3.4.2. SET-PT Mechanism

The SET-PT mechanism is often thermodynamically least likely due to high IP values [44]. However, it is important to note that there exist certain instances when the SET-PT mechanism might exhibit dominance [54,55,56,57,58,59,60]. In methanol, the first step of the SET-PT mechanism for all investigated compounds, which is described by IP values, is extremely endothermic, whereas the second step is significantly exothermic in methanol (Table 5). As anticipated, the IP values were approximately 100 kJ mol^−1^ greater in the non-polar solvent than in methanol. Due to their high values, however, it can be inferred that the SET-PT mechanism is not a viable reaction pathway in either solvent. The theoretically predicted reactivity order was **3f** > **3e** > **3a**, **3c** > **3b** > **3d**. Although the studied compounds had lower and even exergonic PDE values, the initial step of the SET-PT pathway was the limiting factor for the antioxidant activity of these compounds via this mechanism.

#### 3.4.3. SPLET Mechanism

The heterolytic cleavage of the O–H and N–H bonds of investigated compounds is the first step of the SPLET mechanism, which leads to the formation of the corresponding anions. The calculated thermodynamic parameters for the heterolytic cleavage of the O–H and N–H groups are shown in Table 5. The PA values of the O–H group were lower than those of the N–H group. The order of activity based on the PA value for the O–H group was **3d** > **3b** > **3c** > **3a** > **3f**. The difference in reactivity was a consequence of the stabilization of the newly formed anionic species (Figures S3 and S4). According to the PA values in methanol (Table 5), the heterolytic cleavage of the O2′–H bond in **3d** is favourable. The negative charge of this anion is distributed over O7″, O2″, O3″, and C5″. This anion is additionally stabilized with the hydrogen bonds. As expected, the stabilization of the anionic species in the polar solvent was much more pronounced, which is reflected in the lower PA values. Because the BDE values in benzene are lower than the PA and ETE values, it is plausible that HAT predominates in nonpolar solvents. The PA values in methanol were considerably lower than the BDE values; however, the ETE values were higher, but still comparable with the BDEs. Therefore, it is plausible to assume that HAT and SPLET compete within polar media.

### 3.5. Radical Scavenging Activity toward DPPH Radical

**Figure 3:**
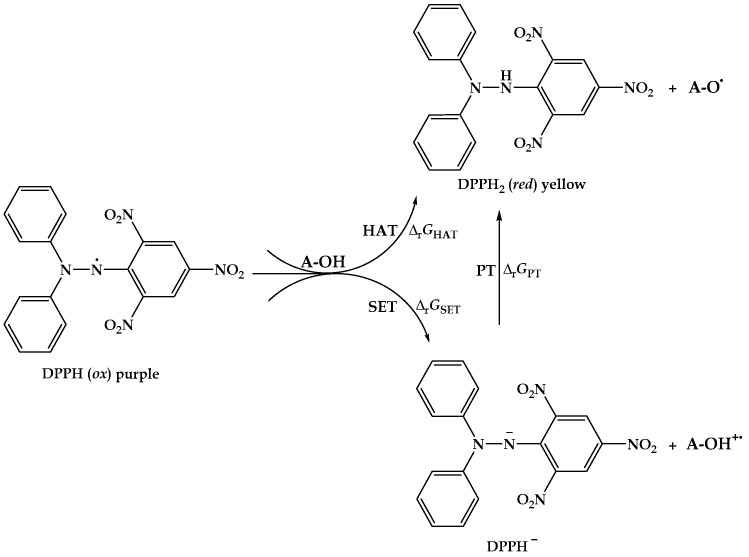
The possible reaction mechanism: **HAT** and **SET-PT**, between DPPH radical and investigation compounds **3a**–**3f** (**A–OH**).

Potential free radical scavenging mechanisms are discussed based on the reaction free energies (Δ_r_*G*) following the HAT and SET-PT mechanisms (Figure 3). Calculations were performed using methanol as the solvent, mimicking the in vitro DPPH test conditions. The difference in energies between the reactants and products serves as the primary criterion for determining the probability of the occurrence of the reaction. The lower value of thermodynamic parameters describing HAT and SET mechanisms indicates the favorability of the pathway by which reaction occurs. The optimized geometries of the reactants involved in the reaction, namely **DPPH^•^**, **DPPH_2_**, and **DPPH^−^** are presented in Figure S5.

An examination of the results presented in Table 6 shows that all the investigated compounds exhibited positive ∆_r_*G*_SET_ values (>132 kJ mol^−1^). This implies that the reactions involving the inactivation of the DPPH radical through the SET-PT mechanism are characterized by endergonic values. Based on the observed data, it can be concluded that the reactivity of the compounds decreased in the following order: **3f** > **3e** > **3a, 3c** > **3b** > **3d**. The significant values of ∆_r_*G*_SET_ clearly demonstrate that the SET-PT mechanism can be disregarded considering DPPH radical scavenging, in agreement with the thermodynamic data presented in Table 6.

Δ_r_*G*_HAT_ values have been identified as a reliable measure for assessing the ability of coumarin derivatives to inactivate DPPH^•^. The observed antioxidant activity, as assessed by in vitro measurements, exhibited the same trend as the Δ_r_*G*_HAT_ values obtained in methanol: **3d** > **3c** > **3f** > **3b** > **3a** > **3e**. This finding further substantiates the notion that both this parameter and spin density possess the capacity to elucidate the reactivity with respect to DPPH^•^. The enhanced antioxidant activity of compounds **3b** and **3f** has been previously highlighted because of the intramolecular stabilization of the radical species produced. Based on the aforementioned information, it is reasonable to anticipate that the DPPH^•^ present in methanol will undergo scavenging via the HAT mechanistic pathway.

### 3.6. Radical Scavenging Activity toward ABTS Radical Cation

**Figure 4:**
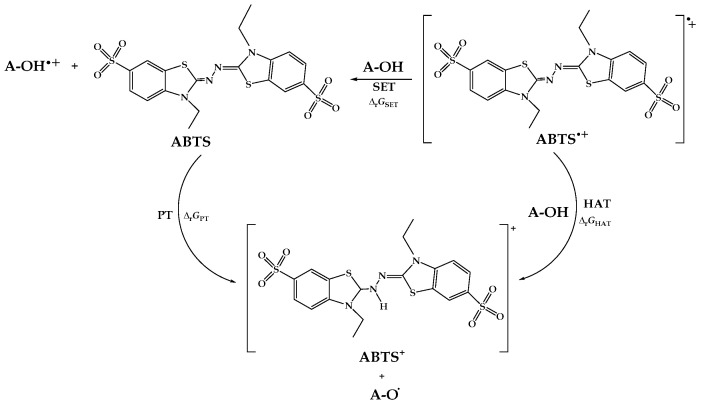
The possible reaction mechanism: **HAT** and **SET-PT**, between **ABTS** radical cation and investigated compounds **3a**–**3f** (**A–OH**).

ABTS^•+^ inactivation was evaluated using two different reaction pathways, namely HAT and SET-PT. Methanol was used as the solvent to replicate the experimental conditions (Figure 4). Table 7 lists the estimated values of the thermodynamic parameters. The optimized geometries of **ABTS^•+^**, **ABTS^+^**, and **ABTS** are shown in Figure S6.

The transfer of a hydrogen atom from the –OH groups of **A–OH** to **ABTS^•+^**, with the formation of **A–O^•^** and **ABTS^+^**, is a thermodynamically favored reaction. The exergonic Δ_r_*G*_HAT_ values of compound **3d** (−13 kJ mol^−1^) demonstrated the highest efficiency in neutralizing radical species, which aligns with the findings from the experimental data. 

On the other hand, electron transfer from **A–OH** to **ABTS^•+^** with the formation of **A**-**OH^+^** and **ABTS^+^** thermodynamics was favored in all the investigated compounds. Based on an analysis of the Δ_r_*G*_SET_ values, it can be concluded that compound **3f** (−155 kJ mol^−1^) exhibits the highest electron transfer ability. However, endergonic Δ_r_*G*_PT_ values (>118 kJ mol^−1^) represent a limiting factor for the manifestation of antioxidant activity through the SET-PT mechanism. Based on the sum Δ_r_*G*_SET_ + Δ_r_*G*_PT_, it can be concluded that the reactivity of the compounds under investigation follows a decreasing trend in the sequence **3d** > **3c** > **3f** > **3b** > **3a** > **3e**, which aligns with the experimental results. In summary, it can be concluded that HAT and SET-PT are in competition when it comes to the inactivation of the **ABTS^•+^** radical in methanol.

## 4. Conclusions

In this study, six coumarin *N*-acylhydrazone derivatives have been synthesized in high yields and characterized using NMR and IR spectroscopy, as well as elemental analysis. Also, the mechanism for the synthesis of coumarin *N*-acylhydrazone derivatives **3** in the presence of molecular iodine as a catalyst was proposed. All the obtained compounds were subjected to investigation of their antioxidant potential using DPPH, ABTS, and FRAP tests. The obtained in vitro results revealed that newly synthesized phenolic compounds **3c** and **3d** exerted excellent activities compared with reference compounds. The Gibbs free energies of reactions were used to determine the most likely mechanism of action. This methodology includes calculations of energy changes during a chemical reaction, providing insight into the feasibility of different reaction pathways. This study also highlighted the importance of considering solvent polarity and free radical species when studying antiradical properties. It was found that HAT and SPLET are viable molecular pathways for the radical scavenging activity of **3a**–**f** in polar mediums, while the HAT is the dominant mechanism in non-polar environments. The changes in Gibbs free energies of reactions suggest the HAT and SET-PT as thermodynamically preferred mechanisms for ABTS^•+^ radical inactivation in methanol. Taking into account all the obtained results in terms of the synthesis and antioxidant potency of the isolated compounds, it can be concluded that significant progress has been achieved in relation to the previous study related to coumarin hydroxy benzohydrazides [26]. Namely, the reactions lasted for much shorter periods, the yields of the isolated compounds were very good, and the antioxidant potential of some of the analogues was better.

## Figures and Tables

**Table 1:** Optimization of reaction conditions.

Entry	Catalyst (mol%)	Yield (%)
1	None	trace ^a,c^/65 ^b,c^
2	CH_3_COOH (10 mol%)	30 ^a,c^/75 ^b,c^
3	CH_3_COOH (20 mol%)	42 ^a,c^/80 ^b,c^
4	I_2_ (5 mol%)	85 ^a,d^
5	I_2_ (10 mol%)	91 ^a,d^
6	I_2_ (20 mol%)	91 ^a,d^

Reaction conditions: **1** (1 mmol), **2a** (1 mmol), 3 mL EtOH; ^a^ Reaction performed at the room temperature; ^b^ Reaction performed by heating under reflux; ^c^ Reaction finished for 10 h; ^d^ Reaction finished for 20 min.

**Table 2:** Isolated yields of coumarin *N*-acylhydrazone derivatives **3**.

Compound	Time (Min)	Yield (%)
**3a**	20	91
**3b**	20	93
**3c**	20	82
**3d**	30	80
**3e**	30	95
**3f**	20	83

**Table 3:** DPPH scavenging activity of products **3** and referent compounds.

Compound	DPPH Scavenging Ability (%)						IC_50_ (µM)	SF
	25 µM		50 µM		100 µM			
	20 min	60 min	20 min	60 min	20 min	60 min		
**3a**	1.0 ± 0.7	1.2 ± 0.7	1.3 ± 1.1	4.2 ± 1.1	1.6 ± 1.3	4.6 ± 1.8	-	-
**3b**	3.5 ± 2.3	3.8 ± 3.0	4.2 ± 0.9	8.6 ± 1.2	5.5 ± 0.9	8.6 ± 0.9	-	-
**3c**	94.2 ± 0.4	94.7 ± 0.9	94.1 ± 2.1	94.5 ± 1.8	98.4 ± 1.2	98.6 ± 1.1	2.1 ± 0.1	6.0
**3d**	97.9 ± 1.7	98.1 ± 0.6	98.3 ± 1.4	98.6 ± 0.8	98.4 ± 0.3	98.7 ± 0.4	3.2 ± 0.1	3.9
**3e**	0.5 ± 0.4	7.4 ± 0.3	1.08 ± 0.3	10.8 ± 1.5	1.4 ± 0.4	35.2 ± 0.1	-	-
**3f**	1.6 ± 0.1	29.2 ± 0.3	3.4 ± 0.3	29.7 ± 2.3	8.7 ± 2.5	31.8 ± 1.7	-	-
NDGA	94.6 ± 0.7	94.6 ± 0.6	94.2 ± 0.7	94.2 ± 0.7	94.5 ± 0.2	94.1 ± 0.7	1.7 ± 0.1	7.4
Quercetin	95.3 ± 0.8	95.1 ± 0.9	96.8 ± 1.0	96.5 ± 0.9	95.1 ± 0.9	95.4 ± 0.8	1.9 ± 0.1	6.6

**Table 4:** ABTS radical cation scavenging activity and ferric ion reducing capacity of products **3** and corresponding referent compounds.

Compound	ABTS Radical Cation Scavenging Activity				Ferric Ion Reducing Capacity
	Scavenging Ability (%)			IC_50_ (µM)	
	25 µM	50 µM	100 µM		% AAa
**3a**	9.7 ± 2.5	13.1 ± 2.3	20.5 ± 2.7	-	137.13
**3b**	15.3 ± 0.9	18.5 ± 1.2	31.1 ± 1.8	-	128.96
**3c**	97.8 ± 1.0	98.1 ± 1.2	99.2 ± 1.0	2.2 ± 0.2	718.41
**3d**	98.4 ± 2.1	98.7 ± 0.7	98.8 ± 1.4	3.0 ± 0.1	510.96
**3e**	8.5 ± 1.2	16.5 ± 1.2	25.4 ± 0.8	-	153.77
**3f**	42.4 ± 1.1	43.2 ± 0.4	53.6 ± 2.0	-	216.34
**Trolox**	97.4 ± 0.2	99.3 ± 0.1	99.5 ± 0.3	1.3 ± 0.1	-

**Table 5:** Calculated thermodynamic parameters (in kJ mol^−1^) of antioxidant mechanisms of investigated compounds **3a**–**f**. DFT calculations were performed at SMD/M06-2X/6-311++G(d,p) level of theory in methanol and benzene as solvents.

Compounds	Position	Methanol					Benzene				
		BDE	IP	PDE	PA	ETE	BDE	IP	PDE	PA	ETE
**3a**	4″–OH	386	524	30	143	411	375	646	143	411	359
	N2–H	403		46	167	404	383		150	429	367
**3b**	2″–OH	378	530	16	124	422	377	659	131	389	400
	N2–H	400		38	149	419	384		138	400	397
**3c**	3″–OH	357	524	1	139	385	346	646	113	402	357
	4″–OH	357		1	131	394	345		112	389	369
	N2–H	403		47	167	404	385		152	427	370
**3d**	2″–OH	347	532	−17	112	403	344	665	92	369	389
	3″–OH	360		−4	154	373	369		117	455	327
	N2–H	401		37	146	423	387		135	394	406
**3e**	N2–H	398	522	45	158	408	385	642	156	430	368
**3f**	4″–OH	365	509	23	144	349	369	664	118	443	349
	N2–H	402		61	167	364	383		132	432	364

**Table 6:** Calculated thermodynamic parameters (in kJ mol^−1^) of antioxidant mechanisms between investigated compounds **3a**–**f** and 2,2-diphenyl-1-picrylhydrazyl (DPPH^•^) radical at SMD/M06-2X/6-311++G(d,p) level of theory in methanol.

Compounds	Position	Mechanisms		
		HAT	SET	PT
		Δ_r_*G*_HAT_	Δ_r_*G*_SET_	Δ_r_*G*_PT_
**3a**	4″–OH	52	147	−95
	N2–H	69		−78
**3b**	2″–OH	44	153	−109
	N2–H	67		−86
**3c**	3″–OH	23	147	−124
	4″–OH	24		−123
	N2–H	69		−77
**3d**	2″–OH	14	155	−142
	3″–OH	26		−129
	N2–H	68		−87
**3e**	N2–H	65	145	−80
**3f**	4″–OH	31	132	−101
	N2–H	69		−64

**Table 7:** Calculated thermodynamic parameters (in kJ mol^−1^) of antioxidant mechanisms between investigated compounds **3a**–**f** and 2,2′-azino*bis*-(3-ethylbenzothiazoline-6-sulfonic acid) radical cation (**ABTS^•+^**) at SMD/M06-2X/6-311++G(d,p) level of theory in methanol.

Compounds	Position	Mechanisms		
		HAT	SET	PT
		Δ_r_*G*_HAT_	Δ_r_*G*_SET_	Δ_r_*G*_PT_
**3a**	4″–OH	25	−140	165
	N2–H	42		182
**3b**	2″–OH	17	−134	151
	N2–H	40		173
**3c**	3″–OH	−4	−140	136
	4″–OH	−3		137
	N2–H	42		182
**3d**	2″–OH	−13	−132	118
	3″–OH	−1		131
	N2–H	41		172
**3e**	N2–H	38	−142	180
**3f**	4″–OH	4	−155	159
	N2–H	41		196

## Data Availability

Data are contained within the article and Supplementary Materials.

## Supplementary Materials

The following supporting information can be downloaded at: https://www.mdpi.com/article/10.3390/antiox12101858/s1. Figure S1: NBO spin distribution for formed O-centered radical species; Figure S2: NBO spin distribution for formed N-centered radical species; Figure S3: NBO charge distribution for formed O-centered anionic species; Figure S4: NBO charge distribution for formed N-centered anionic species; Figure S5: Optimized geometry of radical, neutral and anionic DPPH species at M06-2X/6-311++G(d,p) level of theory in methanol (SMD solvation model); Figure S6: Optimized geometry of radical cation, neutral and cation ABTS species at M06-2X/6-311++G(d,p) level of theory in methanol (SMD solvation model); Table S1: DPPH scavenging activity of products **3c** and **3d**, as well as referent compounds at concentrations close to the IC_50_ value; Table S2: ABTS radical cation scavenging activity of products **3c**, **3d**, and referent compound Trolox at concentrations close to the IC_50_ value.
