# Supplementary material for: Coumarin N-Acylhydrazone Derivatives: Green Synthesis and Antioxidant Potential—Experimental and Theoretical Study
Source: Antioxidants (Basel). 2023 Oct 13;12(10):1858. doi: 10.3390/antiox12101858 (PMC10604617; doi:10.3390/antiox12101858)
Supplement: Supplementary file 1 [file antioxidants-12-01858-s001.zip › antioxidants-2646690-supplementary.pdf]

Supplementary Material

Coumarin *N*-acylhydrazone derivatives: green synthesis and antioxidant potential - experimental and theoretical study

Dušica M. Simijonović [1], Dejan A. Milenković [1,*], Edina H. Avdović [1], Žiko B. Milanović [1], Marko R. Antonijević [1], Ana D. Amić [2], Zana Dolićanin[3], and Zoran S. Marković [1,4,*]

[1]*Department of Science, Institute for Information Technologies, University of Kragujevac, Jovana Cvijića bb, 34000 Kragujevac, Serbia*

[2]*Department of Chemistry, Josip Juraj Strossmayer University of Osijek, Ulica Cara Hadrijana 8A, 31000 Osijek, Croatia*

[3]*Department of Biomedical Sciences, State University of Novi Pazar, Vuka Karadžića bb, 36300 Novi Pazar, Serbia*

[3]*Department of Natural Science and Mathematics, State University of Novi Pazar, Vuka Karadžića bb, 36300 Novi Pazar, Serbia*

**Correspondence: zmarkovic@uni.kg.ac.rs; dejanm@uni.kg.ac.rs*

Contents

^{1}H NMR and ^{13}C NMR spectra of products **3a-f** 2
^{1}H NMR and ^{13}CNMR spectra of **3a** 2
^{1}H NMR and ^{13}CNMR spectra of **3b** 3
^{1}H NMR and ^{13}CNMR spectra of **3c** 4
^{1}H NMR and ^{13}CNMR spectra of **3d** 5
^{1}H NMR and ^{13}CNMR spectra of **3e** 6
^{1}H NMR and ^{13}CNMR spectra of **3f** 7
UV-Vis spectra of products **3a-f** 8
Figure S1. NBO spin distribution for formed O-centered radical species 9
Figure S2. NBO spin distribution for formed N-centered radical species 10
Figure S3. NBO charge distribution for formed O-centered anionic species 11
Figure S4. NBO charge distribution for formed N-centered anionic species 12
Figure S5. Optimized geometry of radical, neutral and anionic DPPH species at M06-2X/6-311++G(d,p) level of theory in methanol (SMD solvation model) 12
Figure S6. Optimized geometry of radical cation, neutral and cation ABTS species at M06-2X/6-311++G(d,p) level of theory in methanol (SMD solvation model) 13
Table S1. DPPH scavenging activity of products **3c** and **3d**, as well as referent compounds at concentrations close to the IC_{50} value 14
Table S2. ABTS radical cation scavenging activity of products **3c**, **3d**, and referent compound Trolox at concentrations close to the IC_{50} value 14

^{1}H NMR and ^{13}C NMR spectra of products **3a-f**

^{1}H NMR and ^{13}CNMR spectra of compound **3a**

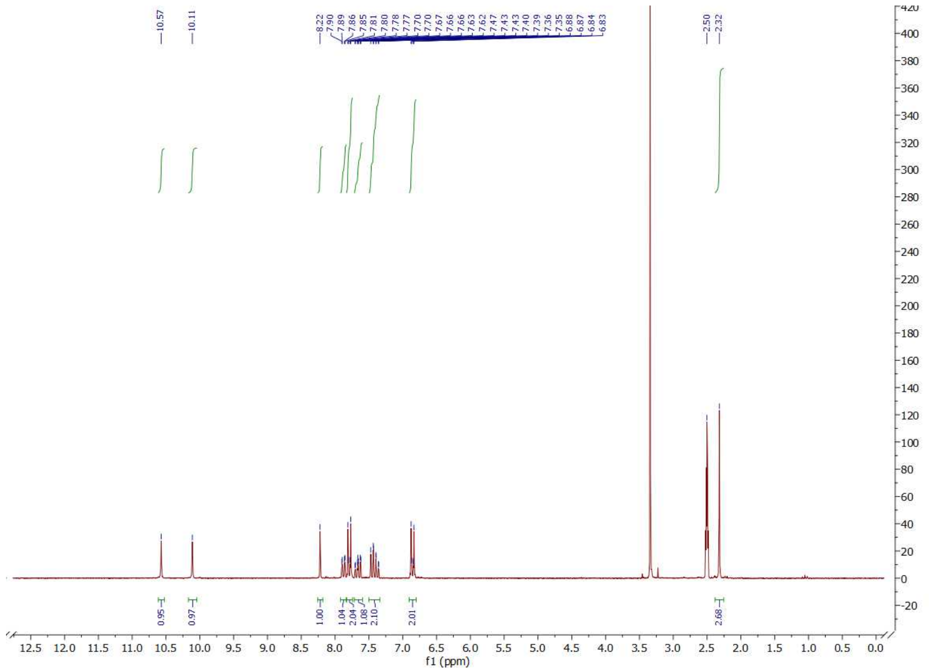

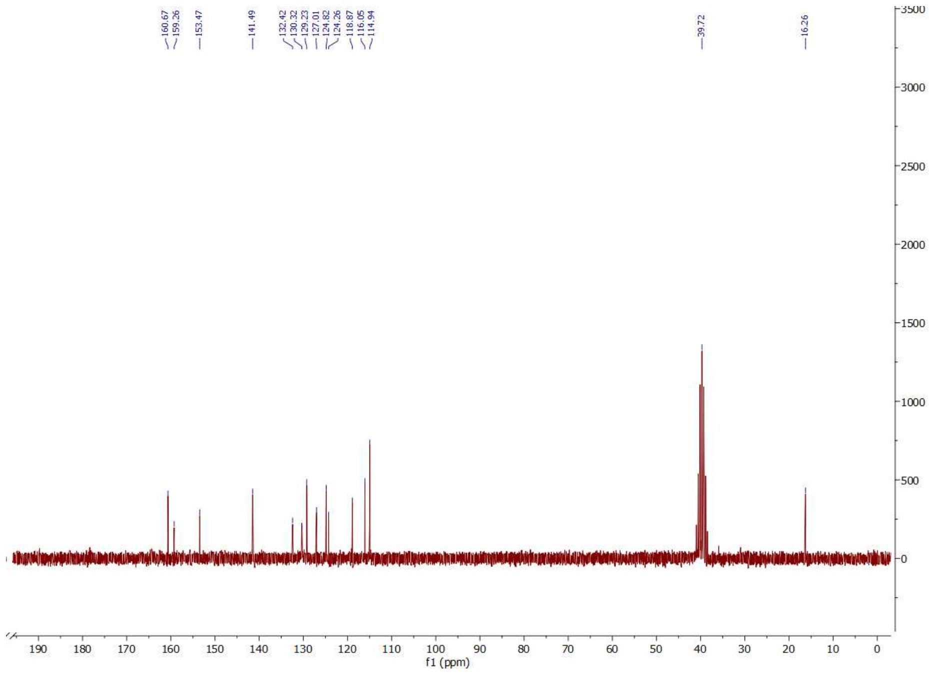

^1H NMR and ^{13}CNMR spectra of compound **3b**

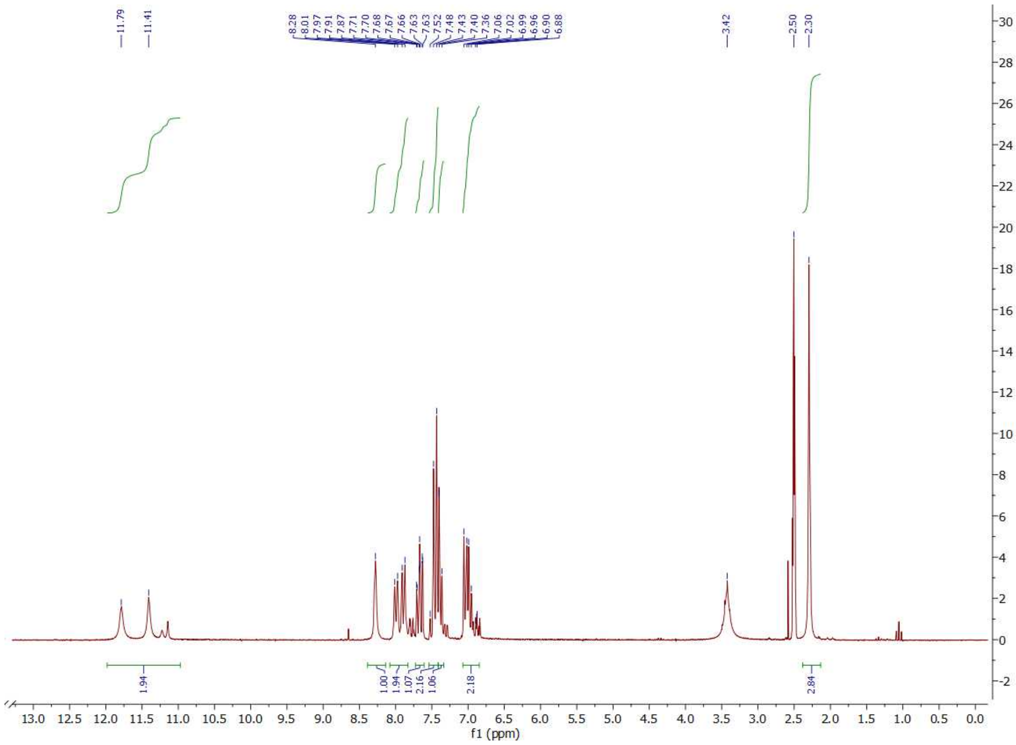

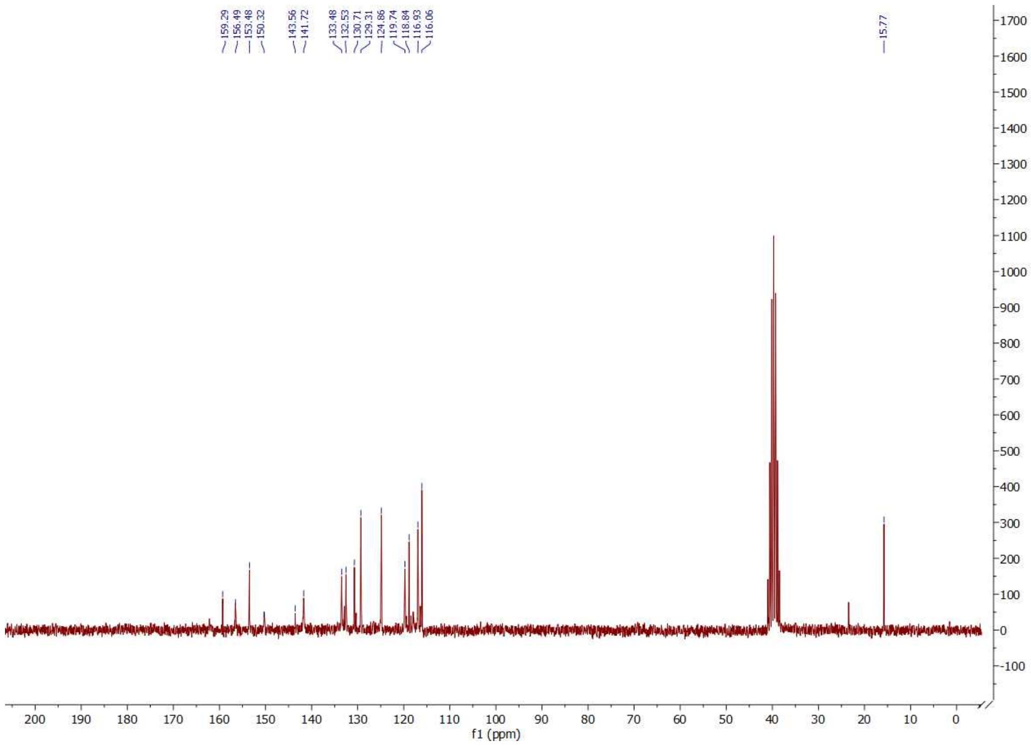

^{1}H NMR and $^{13}CNMR$ spectra of compound **3c**

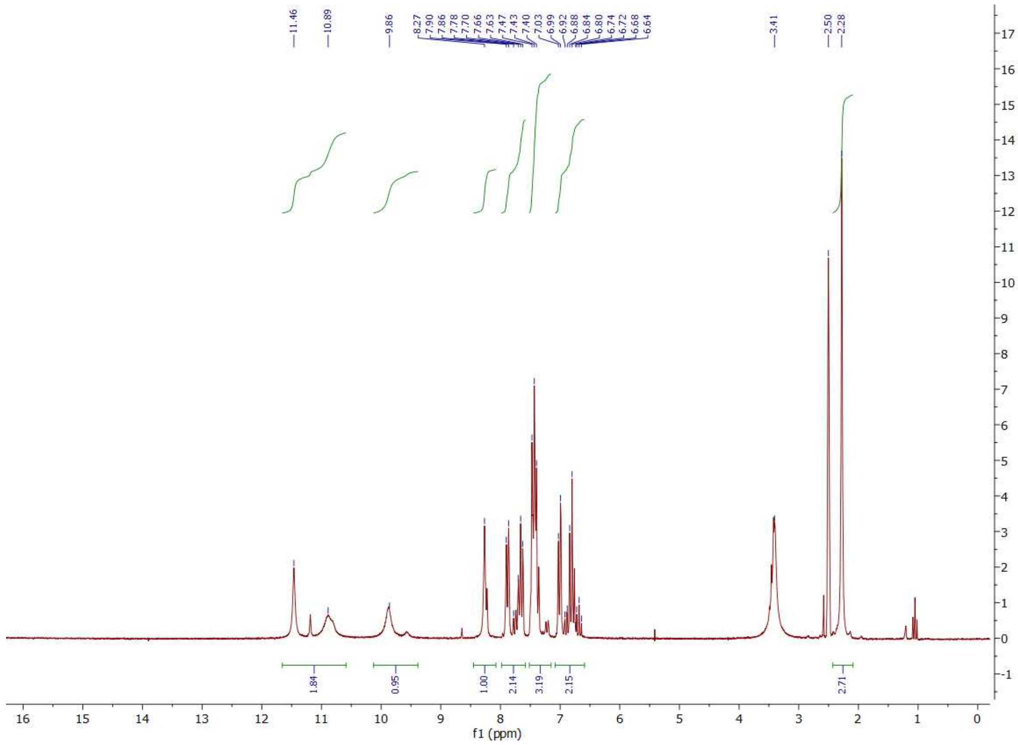

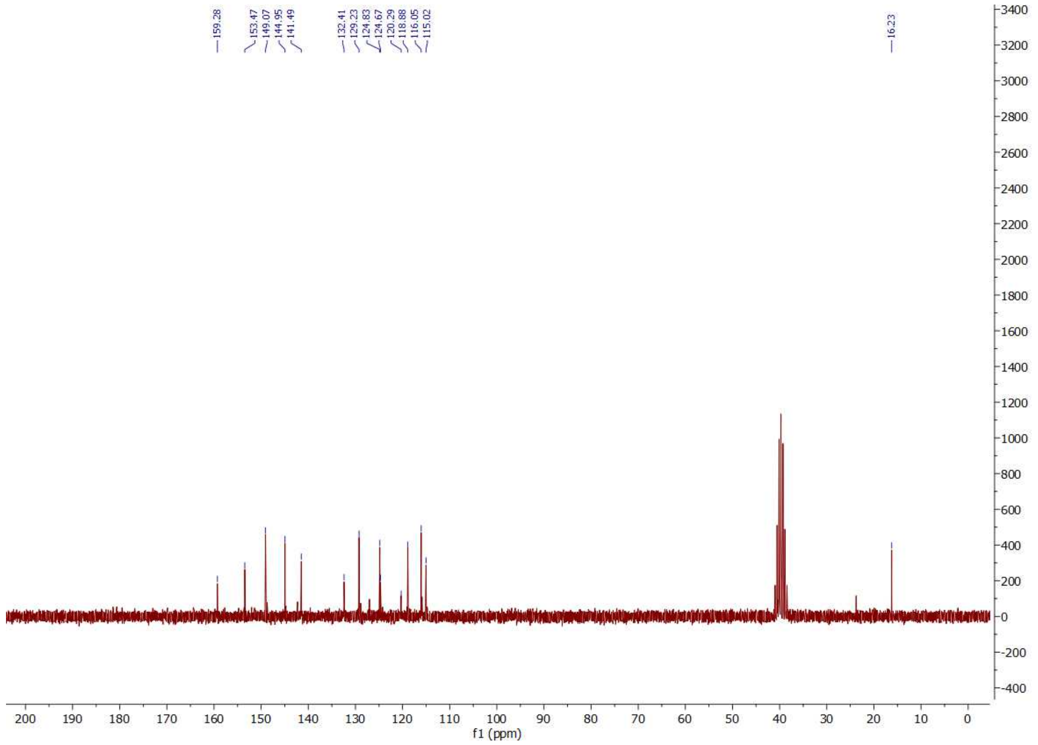

^{1}H NMR and ^{13}CNMR spectra of compound **3d**

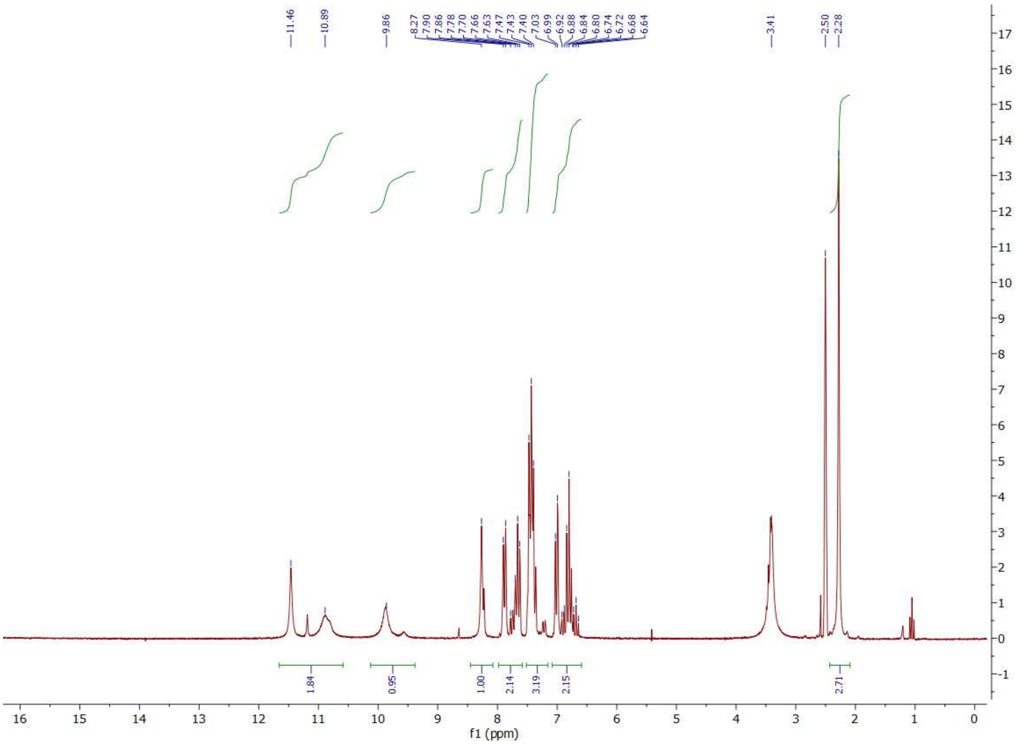

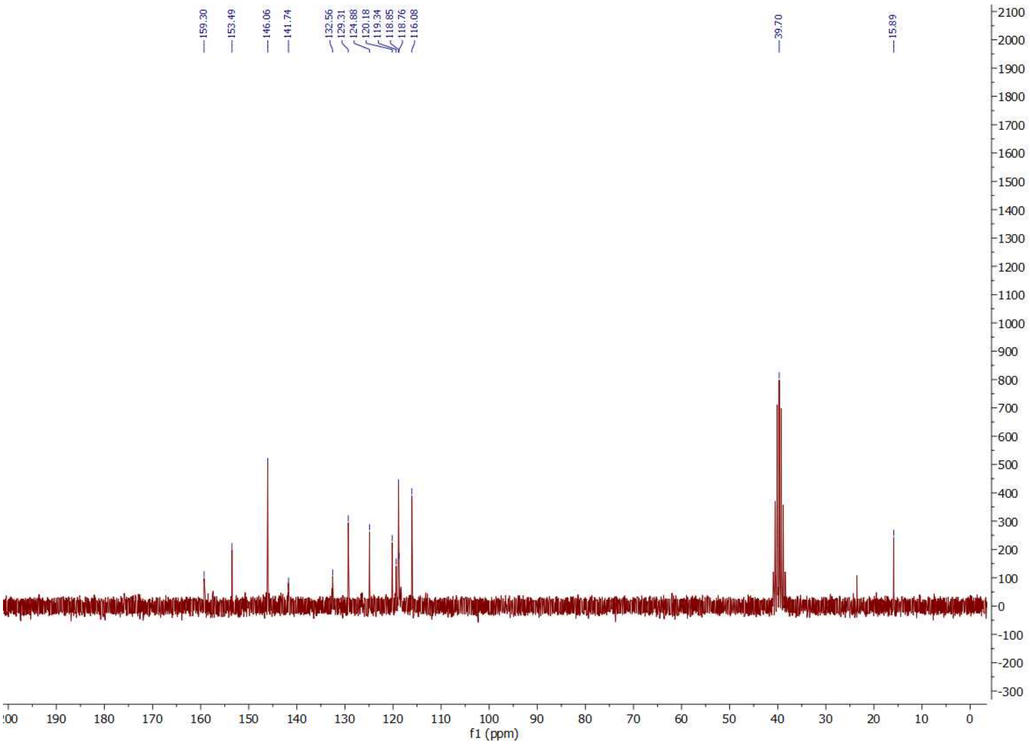

^{1}H NMR and ^{13}CNMR spectra of compound **3e**

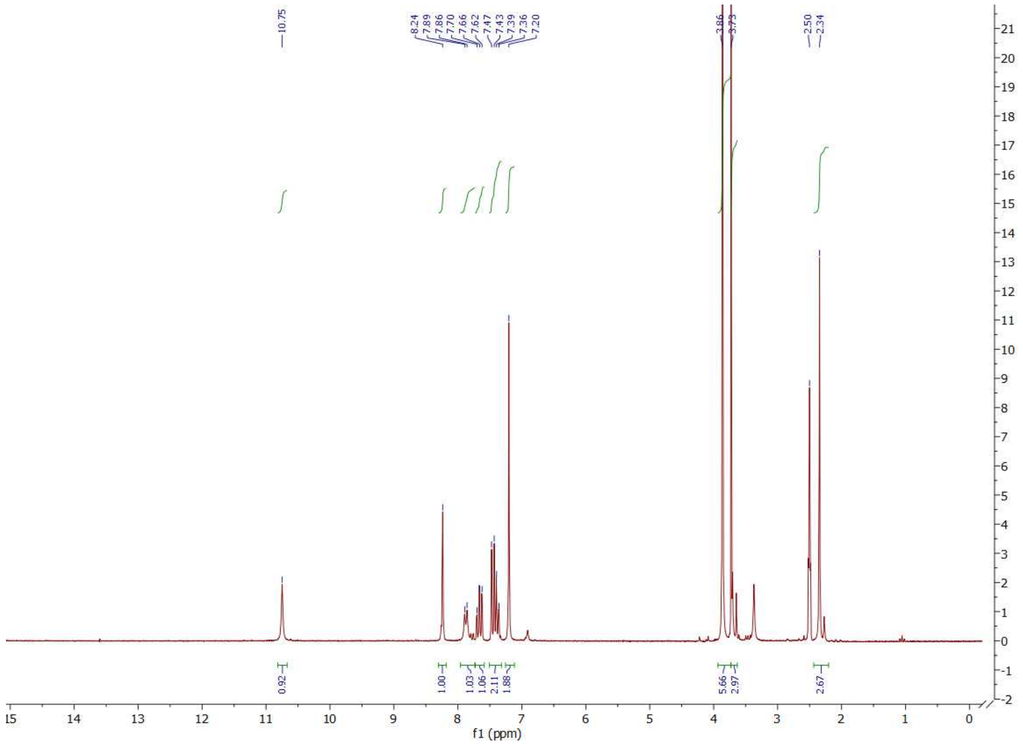

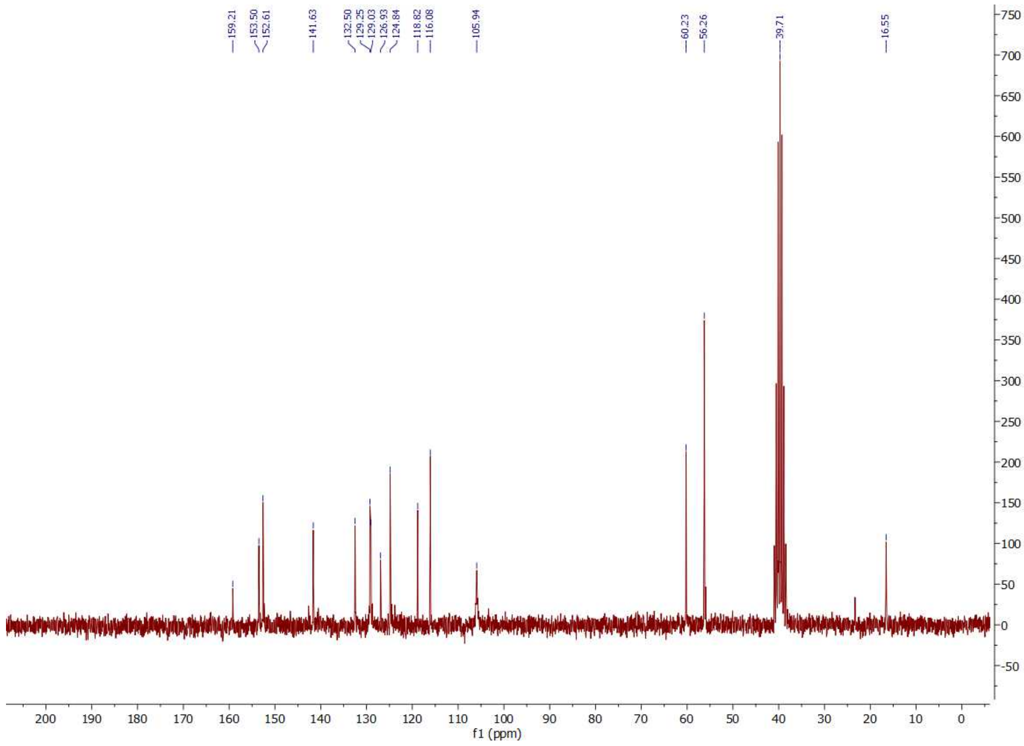

^{1}H NMR and ^{13}CNMR spectra of compound **3f**

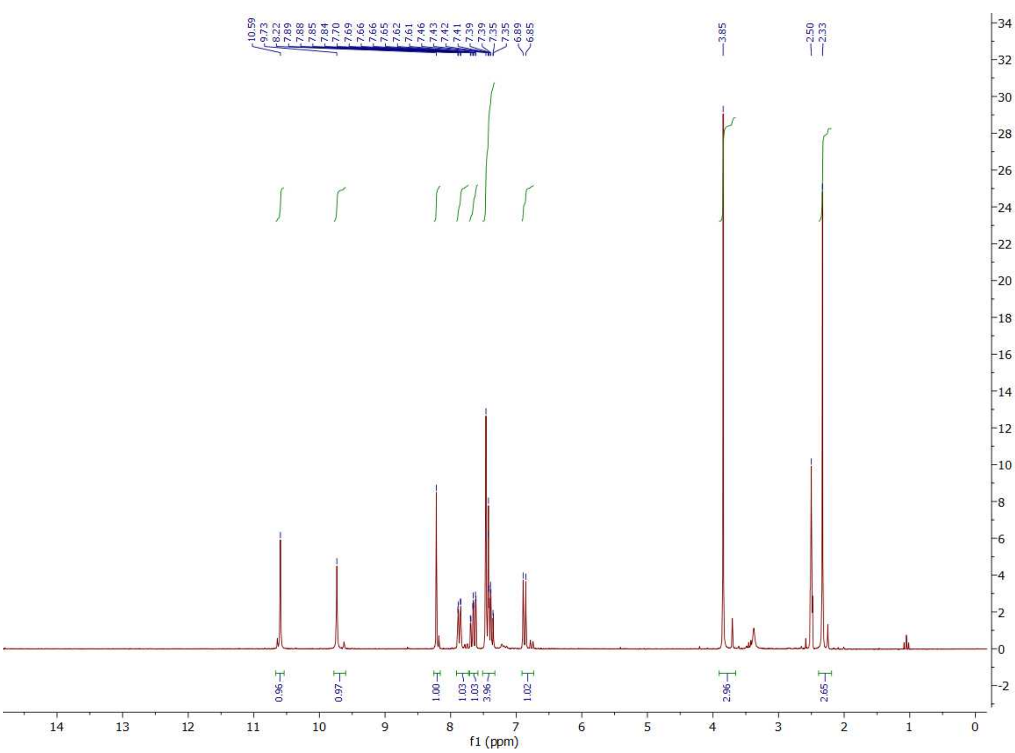

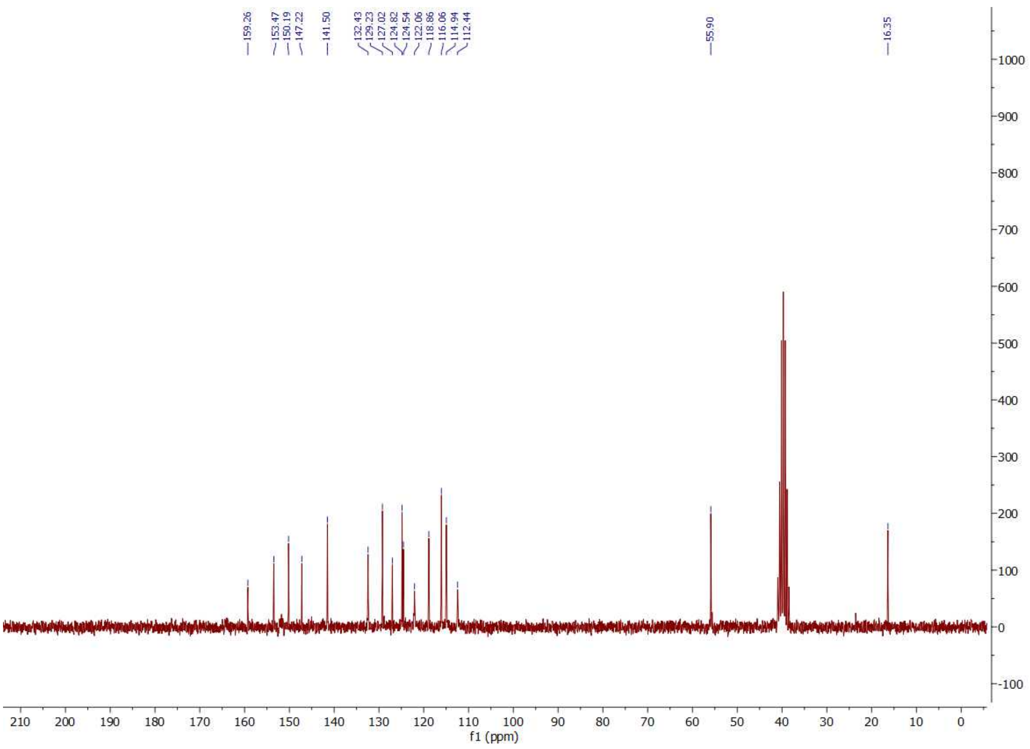

UV-Vis spectra of products **3a-f**

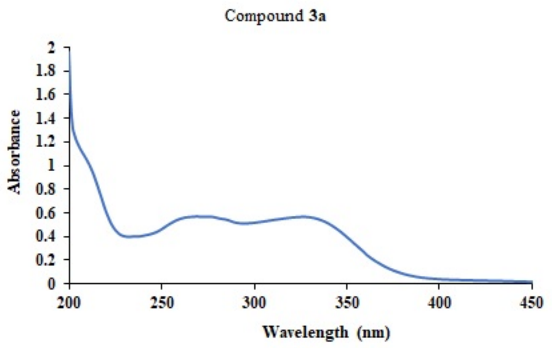

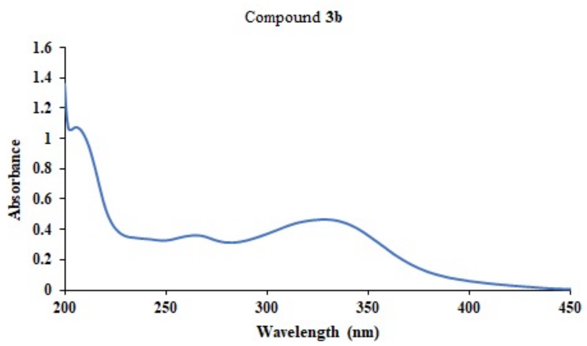

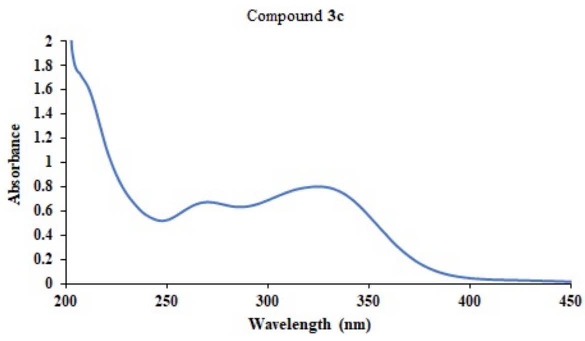

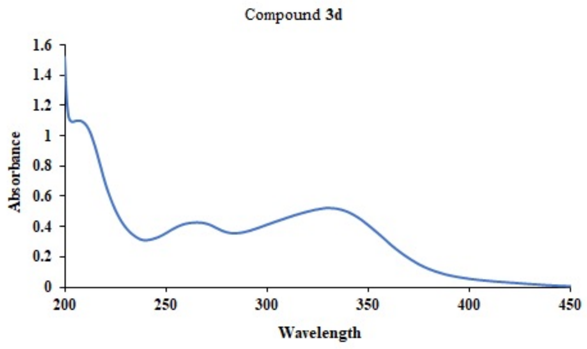

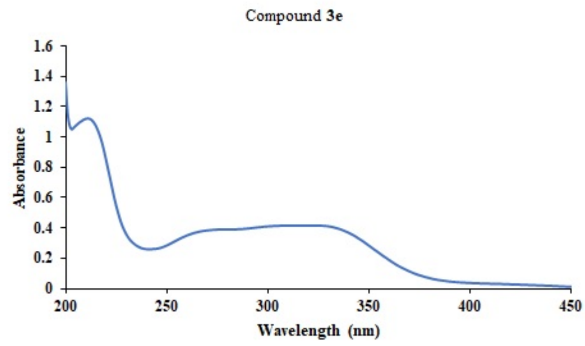

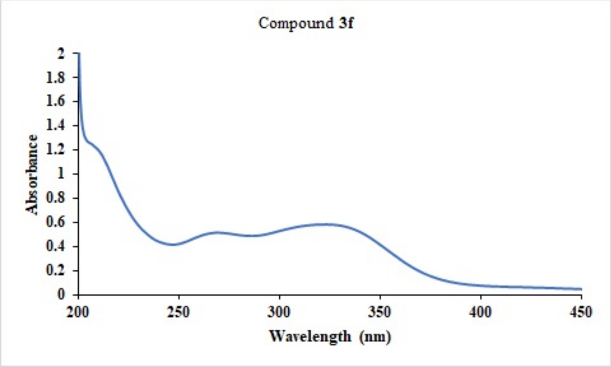

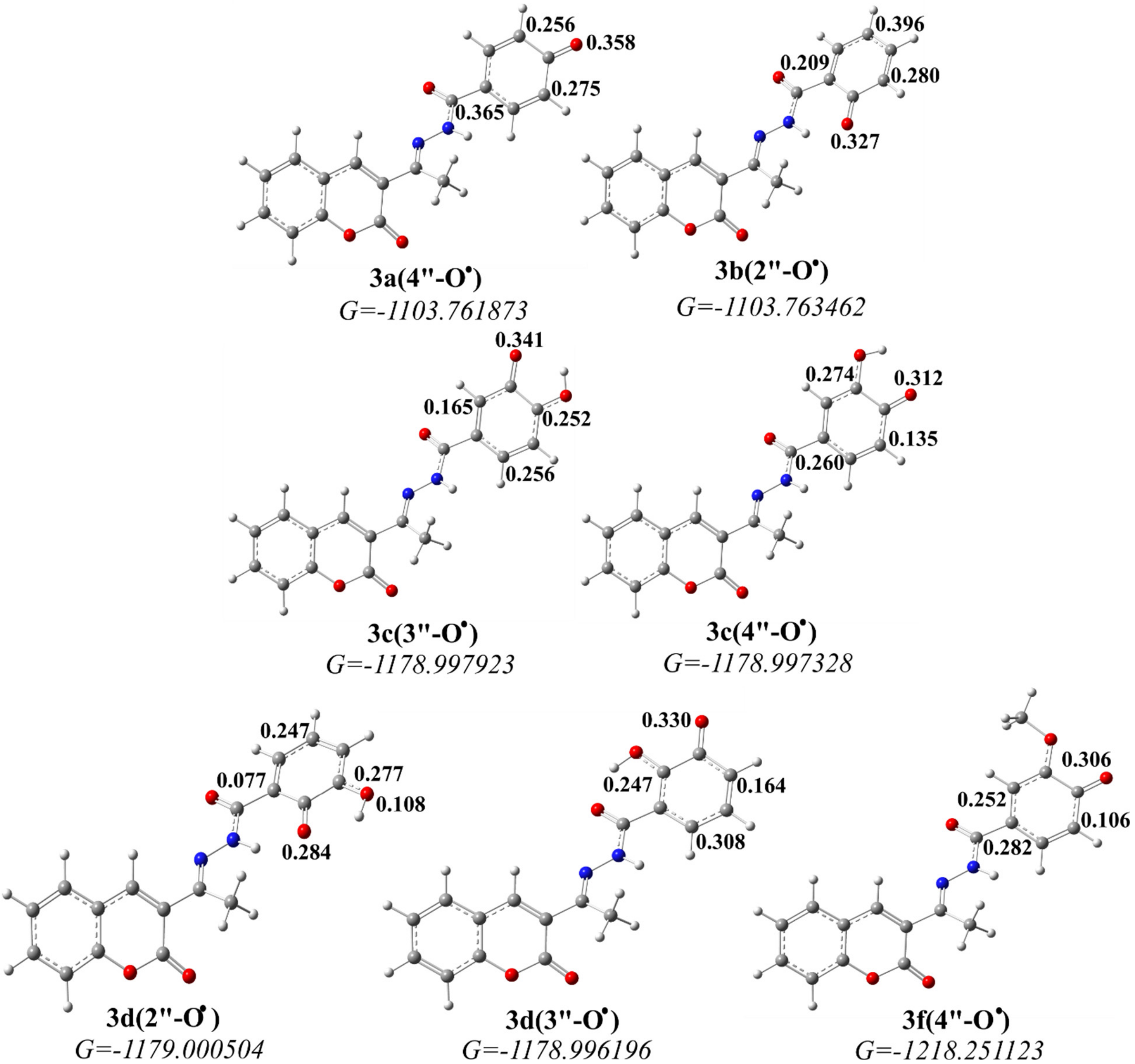

Figure S1. NBO spin distribution for formed O-centered radical species with the values of Gibbs free energy (a.u.)

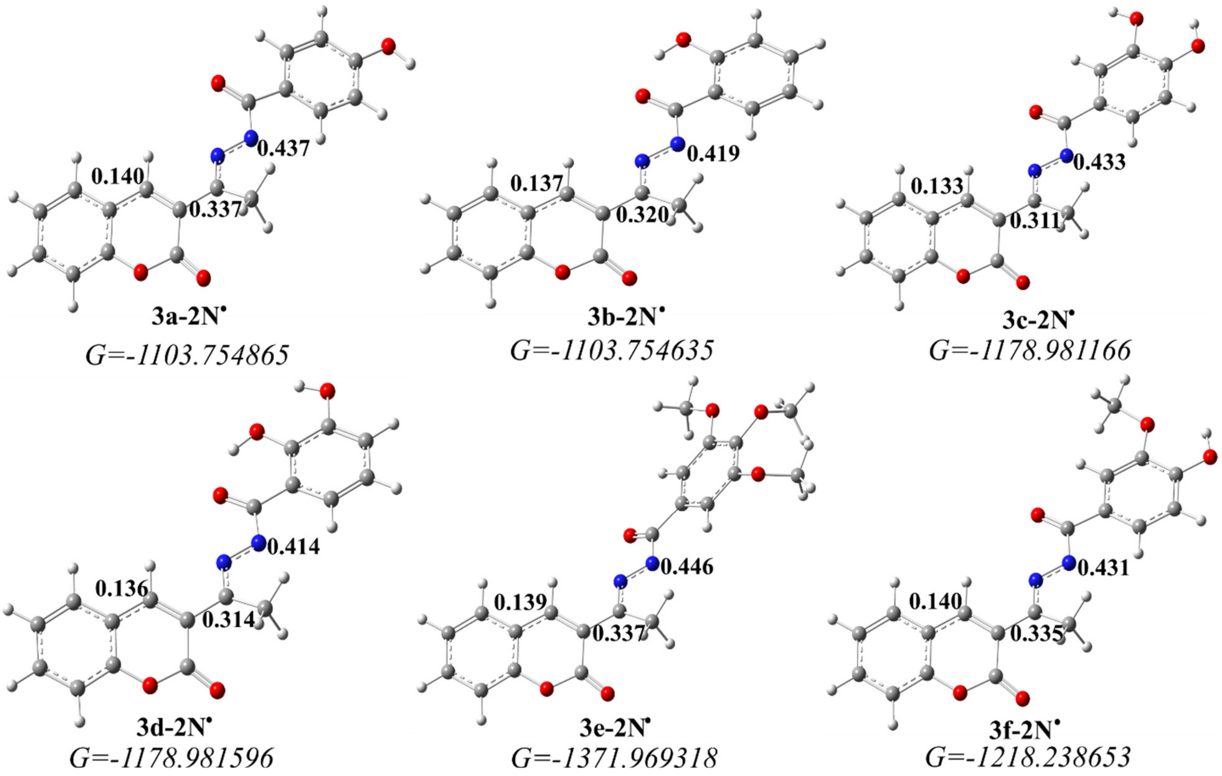

Figure S2. NBO spin distribution for formed N-centered radical species with the values of Gibbs free energy (a.u.)

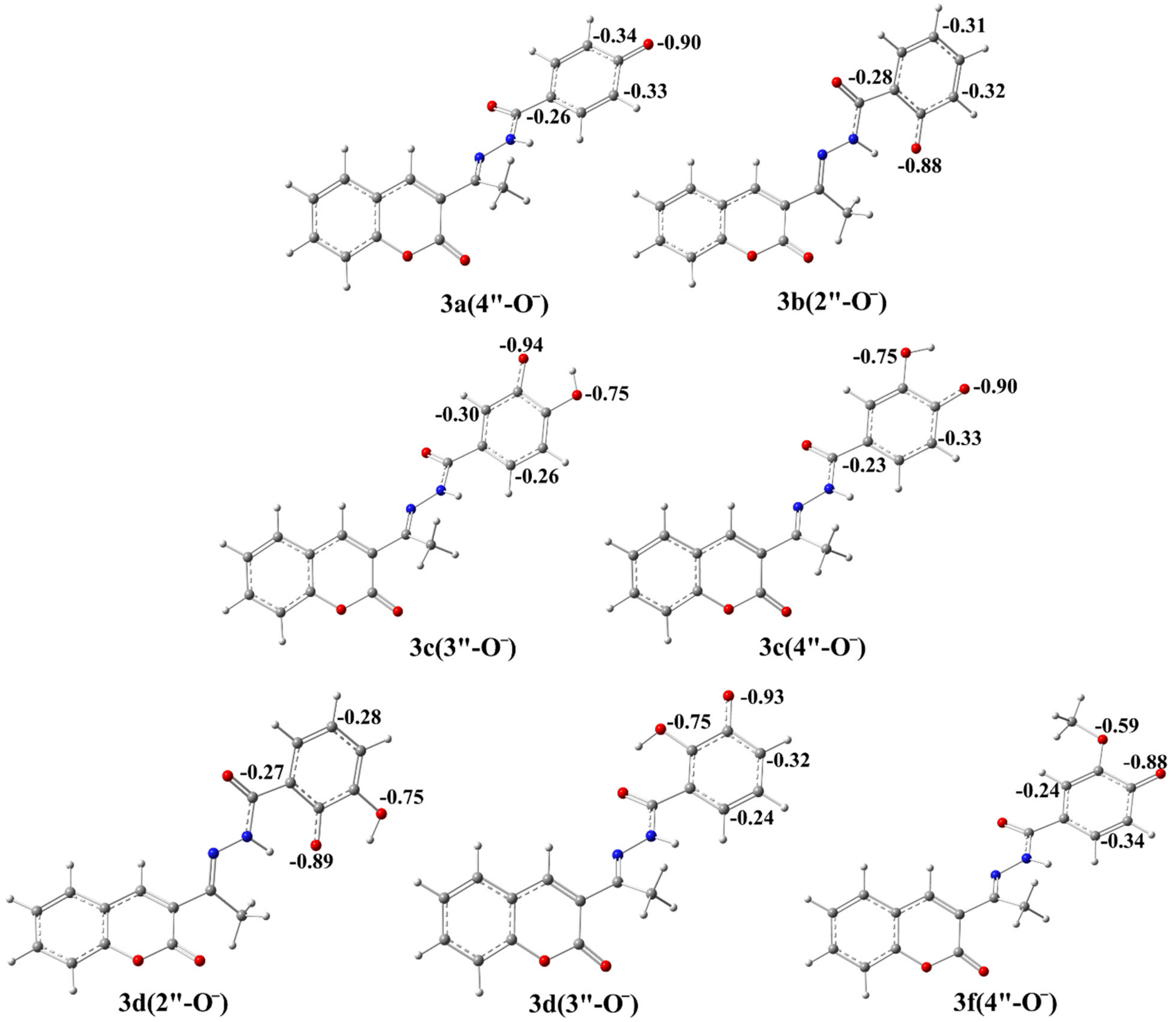

Figure S3. NBO charge distribution for formed O-centered anionic species

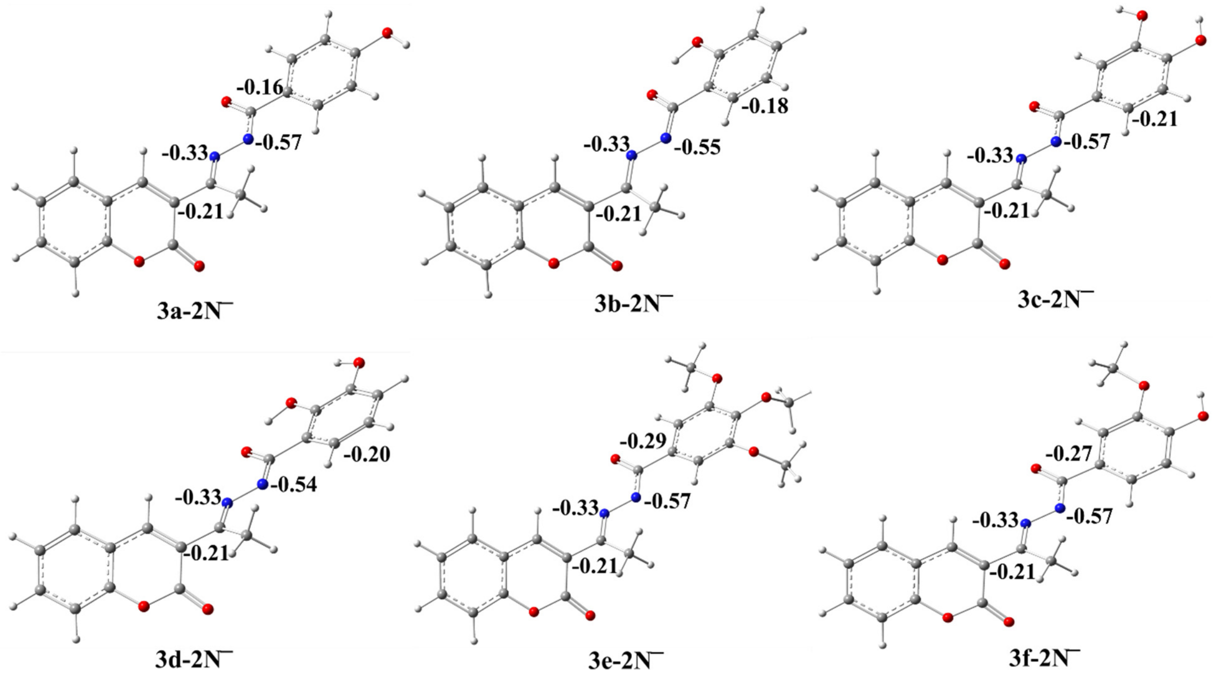

Figure S4. NBO charge distribution for formed N-centered anionic species

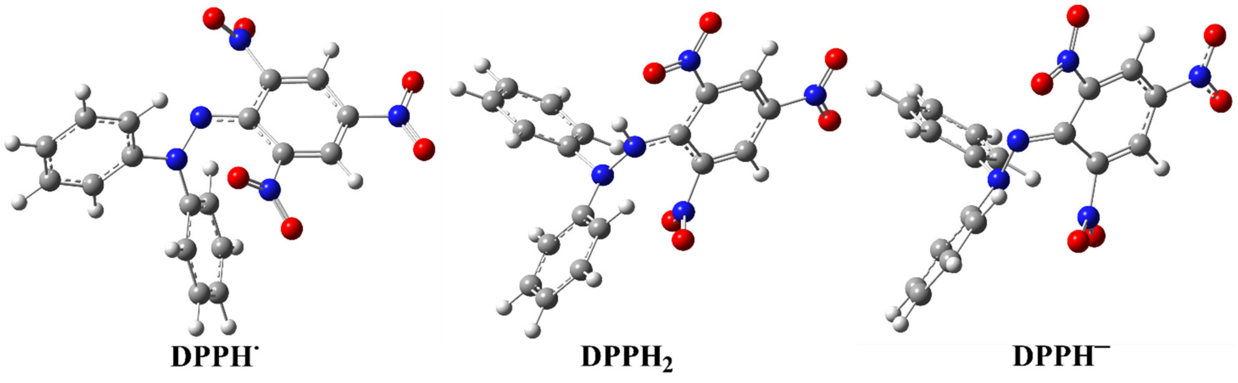

Figure S5. Optimized geometry of radical, neutral and anionic **DPPH** species at M06-2X/6-311++G(d,p) level of theory in methanol (SMD solvation model). Legend: gray-carbon atom, white-hydrogen atom, red-oxygen atom, blue-nitrogen atom.

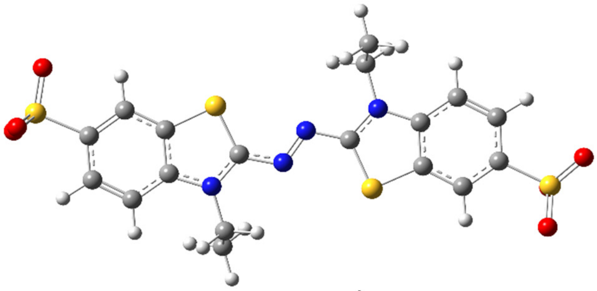

ABTS$^{\bullet+}$

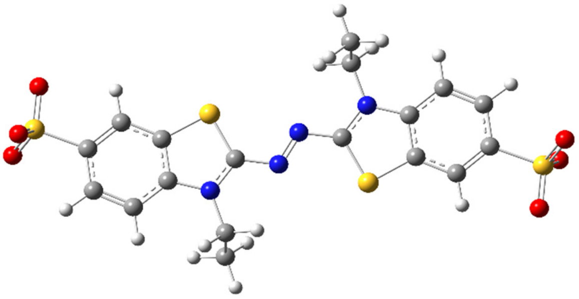

ABTS

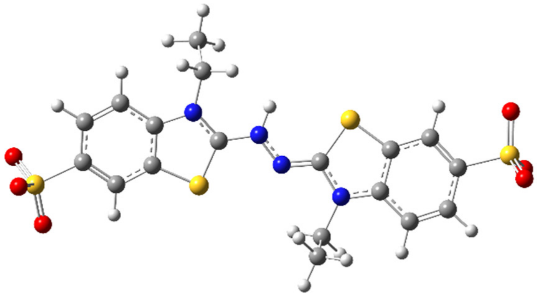

ABTS^{+}

Figure S6. Optimized geometry of radical cation, neutral and cation **ABTS** species at M06-2X/6-311++G(d,p) level of theory in methanol (SMD solvation model). Legend: gray-carbon atom, white-hydrogen atom, red-oxygen atom, blue-nitrogen atom.

Table S1. DPPH scavenging activity of products **3c** and **3d**, as well as referent compounds at concentrations close to the IC_{50} value.

Compound	DPPH scavenging ability (%)					
	1 μM	1.5 μM	2 μM	2.5 μM	3 μM	5 μM
**3c**	16.1	39.3	42.6	64.3	72.1	-
**3d**	-	20.9	26.4	44.7	55.5	74.7
**NDGA**	40.2	42.2	56.3	59.9	79.52	-
**Quercetin**	30.7	45.1	53.9	60.9	77.6	-

Table S2. ABTS radical cation scavenging activity of products **3c, 3d**, and referent compound Trolox at concentrations close to the IC_{50} value.

Compound	ABTS radical cation scavenging activity (%)										
	1 μM	1.5 μM	2 μM	2.5 μM	3 μM	3.5 μM	4 μM	5 μM	6 μM	7 μM	8 μM
**3c**	29.5	39.4	46.4	55.1	65.5	68.8	-	-	-	-	-
**3d**	19.6	27.7	34.5	43.6	52.3	55.4	65.3	77.9	-	-	-
**Trolox**	-	-	-	-	-	-	38.8	48.8	55.3	64.8	71.9
